# Use of Intravenous Immunoglobulin in the Treatment of Pemphigoid Gestationis: A Case Report

**DOI:** 10.7759/cureus.71799

**Published:** 2024-10-18

**Authors:** Julián Martínez-Calvo, Oscar Correa-Jimenez, Alberto Alfaro-Murillo

**Affiliations:** 1 Internal Medicine Department, Hospital San Juan de Dios, San José, CRI; 2 Pulmonology and Immunology in Pediatrics Research Group, School of Medicine, Universidad Nacional de Colombia, Bogotá, COL; 3 Internal Medicine and Clinical Immunology Department, Hospital San Juan de Dios, San José, CRI

**Keywords:** autoimmune bullous disease, intravenous gamma globulin., pemphigoid gestationis, pregnancy, puerperium

## Abstract

Gestational pemphigoid is a rare autoimmune skin condition specific to pregnancy and the postpartum period, with a variable course. There are currently no standardized guidelines referring to evidence-based therapeutic strategies. Intravenous immunoglobulin (IVIG) has recently emerged as a safe and effective steroid-sparing option as a second-line treatment for cases refractory to conventional steroid therapy and for managing relapses. We present the case of a 36-year-old primigravida patient diagnosed with gestational pemphigoid who initially had a difficult clinical course despite treatment with high-dose oral steroids. A first cycle of IVIG combined with steroids (with a gradual dose tapering regimen) was administered, showing initial clinical improvement. However, the patient experienced a relapse that required a new increase in steroid dose and interruption of pregnancy due to fetal growth restriction. Given persistent disease activity at the first outpatient follow-up, a combination of oral steroids with a gradual dose tapering regimen, along with a new cycle of IV immunoglobulin and azathioprine, was initiated, leading to complete resolution by the 19th week of outpatient follow-up. No adverse effects associated with IVIG were reported during the course of follow-up.

## Introduction

Pemphigoid gestationis (PG) is a rare autoimmune blistering disease that affects approximately one in 50,000 pregnancies. It is more common in women with a history of other autoimmune disorders, and it tends to recur in subsequent pregnancies with increasing severity [1].

Clinically, it is characterized by an abrupt onset. The characteristic lesions include papules, urticarial plaques, eczematous lesions, vesicles, and tense blisters that are primarily distributed in the abdomen, immediately or in proximity to the umbilical scar. It may spread or generalize quickly but without affecting mucous membranes. The condition typically arises in the second or third trimester but may present postpartum. Although the disease often resolves after delivery, long-term sequelae, including recurrence in later pregnancies or menstrual cycles, have been documented [2].

This disease is a rare skin condition that belongs to the spectrum of pregnancy and puerperium-specific dermatoses. The pathogenesis of PG is driven by autoantibodies, primarily directed against BP180, a component of the hemidesmosomes that anchor the epidermis to the basement membrane [3].

These autoantibodies activate the complement cascade, leading to an inflammatory response that causes dermoepidermal separation, resulting in blister formation. The histology characteristically shows deposits of C3 and IgG along this structure, a crucial element to confirm the diagnosis [4,5].

Pregnancy represents a challenge in terms of the therapeutic management of autoimmune bullous diseases, considering the safety of the use of currently available pharmacological options. While systemic corticosteroids remain the mainstay of treatment for PG, concerns about fetal growth restriction and maternal complications related to prolonged steroid use during pregnancy have led to the exploration of steroid-sparing therapies. Intravenous immunoglobulin (IVIG) has shown promise in managing autoimmune blistering diseases, offering a safer alternative in refractory cases or in those where corticosteroid-related adverse effects are a concern [6]. We report the safe and effective use of IVIG in the treatment of a pregnant patient with a confirmed diagnosis of PG.

## Case presentation

A 36-year-old female patient, previously healthy, with no personal or family history of autoinflammatory or autoimmune diseases, consulted a tertiary level hospital in week 27 of her first pregnancy, referred by her treating obstetrician-gynecologist with a dermatosis of abrupt onset and at least three weeks of evolution, characterized by vesicles and blisters with involvement of the oral and vaginal mucosa, with extension of the lesions despite treatment with oral deflazacort. The initial dose was 30 mg/day, which was increased after seven days to a dose of 60 mg/day due to poor response to the initially prescribed dose. At the time of hospitalization, the patient provided a biopsy report of the lesions, indicating a diagnosis of classic bullous pemphigoid (BP).

**Figure 1:**
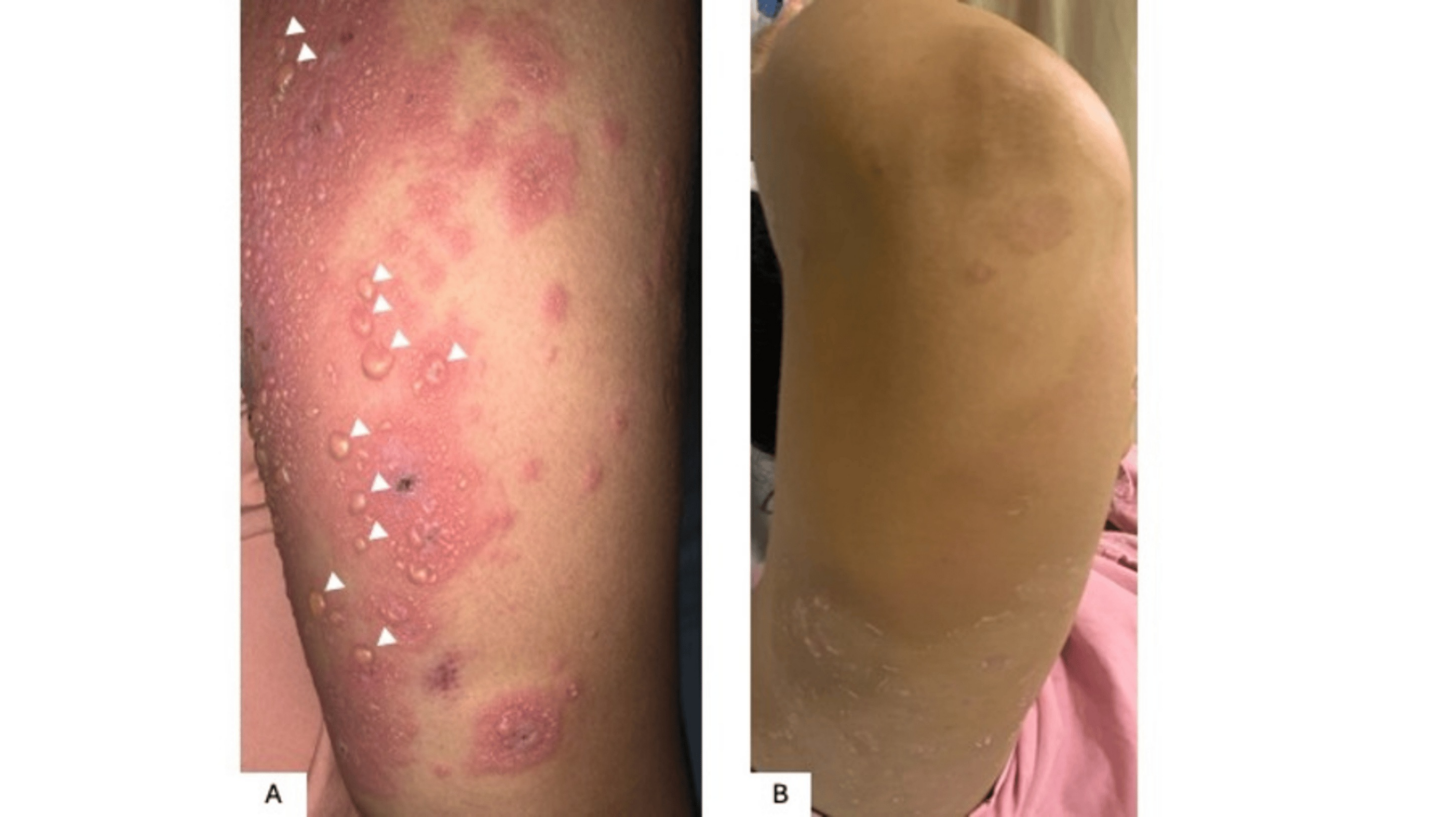
Classic clinical findings of PG: clinical appearance of the skin of the extensor aspect of the right arm of the patient. (A) Multiple confluent urticarial plaques can be observed, on which various tense blisters with serous fluid content are evident. The white arrows highlight the characteristic larger lesions. (B) Complete resolution of the vesicular-bullous eruption is evident after treatment with oral prednisone and IVIG, with areas of hyperpigmentation and thick scaling. PG: pemphigoid gestationis; IVIG: intravenous immunoglobulin.

The initial physical examination revealed generalized dermatosis (facial region, trunk, upper and lower extremities both proximal and distal, palms, and soles), without mucosal involvement, characterized by urticarial plaques with well-defined edges, on which tense vesicles and blisters with serous content developed, without evident erosions at that moment. The lesions were mildly pruritic (Figure 1).

A complete immunophenotype panel was obtained (Table 1). The immunophenotype of lymphocytes exhibited mild T-cell lymphocytosis, primarily due to an increase in CD4+ T cells, which led to a slight elevation of the CD4+/CD8+ ratio. An initial pelvic ultrasound reported a live cephalic product, whose morphology was limited by oligohydramnios.

**Table 1:** Report of the values obtained from the initial immunophenotyping studies of the patient with their respective reference values. The abnormal values are highlighted in bold. *Patients with autoimmune blistering diseases often present an elevated CD4/CD8 ratio, which is associated with disease flares. In this patient, NK cell lymphopenia is potentially related to corticosteroid use or immune modulation during pregnancy. NK: natural killer.

Parameter	Obtained value	Reference value
Total lymphocytes	2,611	915-3,085 cells/uL
CD3 lymphocytes	1,971	500-1,500 cells/uL
CD4 lymphocytes	1,190	370-1,336 cells/uL
CD8 lymphocytes	715	185-1,024 cells/uL
CD4/CD8 ratio*	1.66	0.9-1
CD4 lymphocytes	45.57	30-60%
CD8 lymphocytes	27.39	10-30%
CD4/CD8 double-negative	3	3-10%
CD4/CD8 double-negative count	66	50-200 cells/uL
B lymphocytes (CD19^+^)	595	100-800 cells/uL
B lymphocytes	25	10-20%
NK lymphocytes (CD16 ^+^ CD56 ^+^)*	36	130-730 cells/uL
NK lymphocytes	1.36	5-20%
IgG	800	700-1,600 mg/dL
IgA	179	70-400 mg/dL
IgM	66	40-230 mg/dL
C3	130	90-180 mg/dL
C4	10	10-40 mg/dL

**Figure 2:**
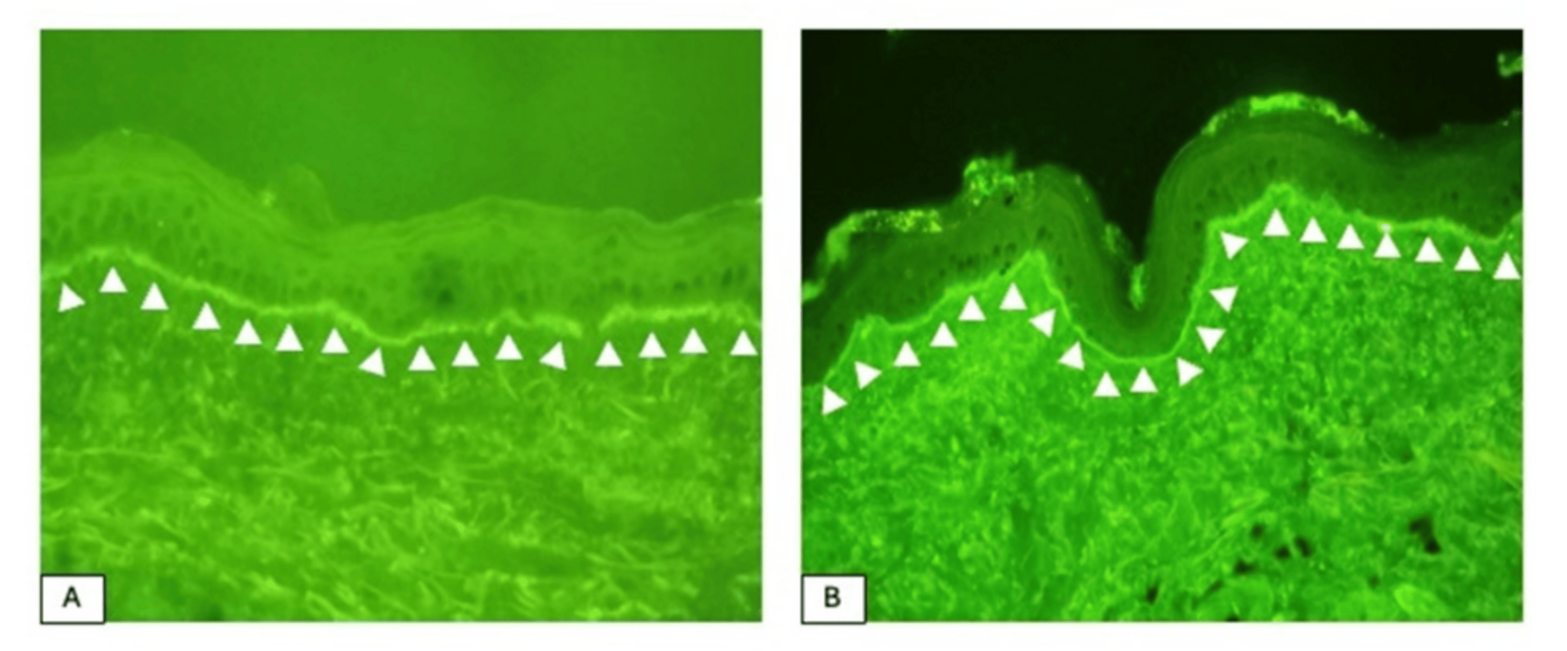
Classic histopathological findings of gestational pemphigoid: Direct immunofluorescence in a skin biopsy sample from the patient's right arm. (A) The white arrows indicate the deposition of the C3 complement fraction in the basement membrane zone, a finding observed in 100% of PG cases diagnosed by biopsy. (B) The white arrows indicate the deposition of immunoglobulin G in the basement membrane zone, a finding observed in 30% of PG cases diagnosed by biopsy. PG: pemphigoid gestationis.

A new skin biopsy from the patient’s right arm showed findings compatible with PG. The deposition of IgG1 and C3 in the basement membrane zone was demonstrated by direct immunofluorescence preparations (Figure 2).

Given the initial therapeutic failure with oral steroids, it was decided to change deflazacort for intravenous methylprednisolone in pulses of 250 mg/day for three days. Subsequently, oral prednisone was started at a dose of 40 mg/day for 14 days, with a gradual decrease in the dose over the following weeks. IVIG was concomitantly administered at a total dose of 2 g/kg administered for five days. Furthermore, antimicrobial prophylaxis with trimethoprim-sulfamethoxazole was administered three times a week. 

A follow-up evaluation after the application of IVIG showed an adequate response to treatment with the absence of new lesions and healing of erosions; therefore, the patient was discharged on the 10th day of hospitalization, continuing with oral steroid tapering therapy.

At the first re-evaluation in the immunology outpatient clinic, eight weeks after discharge, it was documented that the patient presented a relapse of the disease when the prednisone dose was reduced to 15 mg/day, which led to new hospital admission and an increase in the steroid dose. An elective cesarean section was scheduled following the diagnosis of intrauterine growth retardation. The surgery was performed without complications. The newborn was hospitalized for two weeks due to low birth weight. Finally, the newborn was discharged without complications.

Since disease activity persisted and high doses of steroids were required, it was decided to start steroid-sparing treatment with azathioprine (150 mg/day) in association with a new cycle of IVIG. She was reevaluated 19 weeks later, and complete remission of the clinical picture was documented. No adverse effects related to the use of IVIG were described during the patient's follow-up. Breastfeeding was not restricted.

## Discussion

PG is a rare clinical condition that usually presents in late pregnancy, the puerperium, or could be related to gestational trophoblastic disease. This condition usually resolves with delivery. Nevertheless, its clinical course can be chronic, characterized by relapses and exacerbations during the puerperium or related to menstruation or the use of oral contraceptives [5,7]. The disease carries significant morbidity for the pregnant patient and the fetus, manifested predominantly as intrauterine growth restriction, preterm delivery, and low birth weight [4]. This case followed the classic course described with the appearance of typical lesions around 24 weeks of gestation, with an evolution of rapid dissemination and fetal involvement documented by ultrasound and the presence of oligohydramnios, despite the administration of oral corticosteroids.

As is clear, establishing an accurate diagnosis has a direct impact on maternal-fetal treatment and prognosis, as well as guiding the planning of potential future pregnancies. Within the differential diagnosis, the main considerations include BP, another autoimmune blistering disease that, like PG, is characterized by the appearance of eczematous lesions, urticarial lesions, or tense blisters, with mucosal involvement in 10-20% of cases, mediated by the deposition of autoantibodies against BP 180 and 230, and primarily characterized by the linear deposition of IgG and C3 in the area of the basement membrane; and polymorphic eruption of pregnancy, a common, benign inflammatory condition that usually affects primigravida patients, mainly during the third trimester, lacking an autoimmune background, characterized by the appearance of erythematous and pruritic papules within the abdominal striae, and which, in half of the cases, presents polymorphic characteristics such as eczematous plaques, vesicles, or atypical target lesions [8].

Establishing the accurate diagnosis solely based on the clinical appearance of the lesions can be quite challenging, especially in cases of urticarial PG without blisters, which is why immunofluorescence techniques, immunoblotting, or enzyme-linked immunosorbent assay (ELISA) with immunofluorescence should be used [9].

Similarly, there is currently no biomarker that has been shown to be useful in monitoring cases once therapy has been initiated and for predicting the risk of exacerbations. A promising diagnostic technique involves the determination of BP-180NC16a IgG titers using ELISA, allowing for differential diagnosis with polymorphic eruption of pregnancy; however, it should be noted that to date, for PG, there is no utility of the technique for monitoring, as no correlation has been documented between antibody titers and the activity and severity of the disease, as well as with the intensity of immunofluorescence [9].

There is no current consensus on the treatment of this disease. However, Genovese et al. proposed a treatment algorithm that highlights IVIG as a valid option in recalcitrant cases, either in monotherapy or in combination with steroids [5,10]. The treatment of our patient, in addition to IVIG, was carried out with intravenous steroid pulses, followed by a transition to oral administration with the corresponding gradual tapering of the dose. The intention of allowing the rapid tapering of the steroid dose was to avoid the possible complications described for this group of drugs on maternal-fetal health, such as impairment of placental growth with consequences on nutrition and gas exchange with the fetus [11].

The steroid selected for this case was prednisone because it is metabolized by the placental enzyme 11-beta-hydroxysteroid dehydrogenase. This process inactivates prednisolone through its conversion to prednisone, thereby protecting the fetus from elevated prednisolone levels. This mechanism minimizes the steroid's passage to the fetus via the placenta, unlike other steroids not metabolized by this enzyme [12,13].

IVIG contains a concentrate of neutralizing antibodies obtained from healthy donors manufactured according to a strict standardized quality and safety process. Its widespread use is limited mainly by its high cost. The in vivo mechanism of action remains unknown; however, based on in vitro models, it is postulated that the Fc region of IgG induces changes in signal transduction pathways in cells expressing the Fcγ receptor, thus blocking the initiation of the anti-inflammatory cascade and reducing proinflammatory signals, which inhibits antibody production. In addition, IVIG can cross the placenta, providing an additional advantage in the treatment of various autoimmune diseases during pregnancy [14].

In the case of blistering diseases, it is hypothesized that the principal mechanism involves saturation of the neonatal Fc receptor (FcRn). In various murine models and under normal conditions, FcRn has been demonstrated to prevent the pinocytosis and subsequent lysosomal degradation of IgG isotype immunoglobulins, including pathogenic antibodies. This action accelerates the destruction of these antibodies. Additionally, FcRn has been shown to reduce circulating levels of the pro-inflammatory cytokine IL-6 [14].

IVIG is one of the most widely used options as a steroid-sparing agent in the treatment of autoimmune bullous diseases of pregnancy, including PG, with proven efficacy and safety. It accounts for up to 22.2% of the therapeutic strategies used in a recent meta-analysis, including monotherapy. It has also been shown that IVIG does not increase the risk of infections and is safe for the fetus [5].

This clinical case demonstrated a prompt response to the IVIG treatment in combination with corticosteroids, with the cessation of the appearance of new lesions and healing of the previously documented erosions. Nevertheless, it is an example of the clinical course characterized by flares, as the patient again showed signs of disease activity with the appearance of new lesions after reaching the dose of 15 mg/day of oral prednisone, which was severe enough to justify a further increase in the dose of prednisone and to schedule an elective cesarean section after documenting data of fetal morbidity.

For severe cases refractory to treatment with topical or systemic steroids (considered the cornerstone therapy in mild to moderate cases or during pregnancy), or when there is a contraindication for their use, the use of other steroid-sparing agents than IVIG, including azathioprine and dapsone, has been reported. According to a recent systematic review, azathioprine and dapsone are the second and third most frequently used agents after IVIG, preferably administered after the completion of pregnancy, although they may be considered in refractory cases in pregnant patients as a last resort. The safety profile during pregnancy and lactation is limiting for recommending their widespread use: In the case of azathioprine, it is important to monitor the complete blood count (especially during the third trimester of pregnancy) as there is a risk of developing leukopenia, and it should be consumed at least four hours before breastfeeding to minimize the risk of toxicity to the infant. Regarding dapsone, it is highly advisable to rule out glucose-6-phosphate dehydrogenase deficiency in both the mother and the infant before initiating treatment, given the risk of hemolysis [5]. 

It should also be mentioned that cyclosporine and rituximab emerge as fourth-line agents. The latter has even been used anecdotally between six and 12 months preconception, as it is believed to reduce the risk of recurrence in subsequent planned pregnancies [5].

In this case, a new flare of disease activity emerged during the follow-up in the postpartum period. There is no consensus regarding therapeutic options during the postpartum period or lactation; however, the American College of Rheumatology considers the use of corticosteroids (topical or systemic), azathioprine, IVIG, plasmapheresis, and immunoadsorption to be compatible. There are no safety studies for the use of other therapies in this scenario (such as rituximab or tumor necrosis factor-alpha antagonists). The combination of triple therapy with oral steroids, IVIG, and azathioprine as a third line of treatment was favorable, finally achieving clinical remission at week 19 of outpatient follow-up [5,14].

It is important to highlight that the subsequent treatment with IVIG resulted in a reduction in CD4+ T lymphocyte counts and normalization of the CD4+/CD8+ ratio observed during the follow-up. These observations suggest that monitoring T-lymphocyte levels could be effectively used as a follow-up parameter. Nonetheless, there is a notable paucity of studies providing normative values for various T lymphocyte populations in pregnant women. The evaluation of the CD4/CD8 T-cell ratio is critical in autoimmune diseases for both diagnostic assessment and disease monitoring. This ratio reflects the balance between helper (CD4+) T cells and cytotoxic (CD8+) T-cell populations, which are crucial for immune regulation. In autoimmune diseases, such as multiple sclerosis, systemic lupus erythematosus, and rheumatoid arthritis, an altered CD4/CD8 ratio is often observed, indicating immune dysregulation [13,14].

Several studies have demonstrated that an increased CD4/CD8 ratio is strongly associated with autoimmune diseases, including autoimmune blistering skin disorders such as BP and pemphigus vulgaris (PV). Specifically, it has been shown that patients with PV exhibit a significantly elevated CD4/CD8 ratio compared to healthy individuals, and this increase correlates with disease activity [15].

Moreover, it has been evidenced that an increased CD4/CD8 ratio in patients with BP is associated with disease flares and worsening of skin lesions. It has also been reported that immunosuppressive treatment may help normalize the CD4/CD8 ratio as clinical symptoms improve, suggesting that this ratio could serve as a valuable biomarker for assessing treatment response [16].

In relation to the low NK cell levels observed in the patient, it is important to highlight that this decrease may be secondary to corticosteroid use. However, it has been well-documented that pregnancy itself is associated with alterations in immune cell populations, including a reduction in natural killer (NK) cells. These immunological shifts are thought to promote fetal tolerance by modulating the maternal immune response, thereby preventing excessive immune activation. Specifically, decreased NK cell activity during pregnancy has been reported, potentially contributing to the creation of an immunologically favorable environment for fetal development. These findings suggest that the suppression of NK cells represents a physiological adaptation during pregnancy, aimed at ensuring successful gestation while preserving immune homeostasis [17].

Finally, it is important to emphasize the clear benefit of a multidisciplinary approach in the early management of a suspected case of gestational pemphigoid, with the aim of reducing the maternal-fetal morbidity and mortality associated with the disease. This involves collaboration between the obstetrician, dermatologist, and immunologist, which facilitates timely diagnosis, treatment, and follow-up [4,5].

## Conclusions

There is currently no consensus to guide therapeutic strategies or patient follow-up in PG. Current evidence comes from systematic reviews and meta-analyses that demonstrate the efficacy and safety of IVIG as a second-line option in combination with steroids, or even as monotherapy, with the limitation of its high cost, which makes its generalized use difficult. This case report demonstrated a favorable response to such treatment during pregnancy and in the setting of relapse of disease activity in combination with oral steroids and azathioprine. There is a compelling need for further research on IVIG management strategies for PG to strengthen the evidence collected so far, which may eventually facilitate the development of a valid and protocolized treatment consensus. It will also be important to develop follow-up strategies that allow for the assessment of therapeutic response and prediction of the likelihood of recurrence in this disease in conjunction with the clinical evolution.
